# Ubiquity of anomalous transport in porous media: Numerical evidence, continuous time random walk modelling, and hydrodynamic interpretation

**DOI:** 10.1038/s41598-019-39363-3

**Published:** 2019-03-14

**Authors:** Xiao-Rong Yang, Yan Wang

**Affiliations:** 1 Tibet University, School of Science, Lhasa, 850000 China; 2 Iowa State University, Department of Statistics, Ames, Iowa 50011 USA

## Abstract

Anomalous transport in porous media is commonly believed to be induced by the highly complex pore space geometry. However, this phenomenon is also observed in porous media with rather simple pore structure. In order to answer how ubiquitous can anomalous transport be in porous media, we in this work systematically investigate the solute transport process in a simple porous medium model with minimal structural randomness. The porosities we consider range widely from 0.30 up to 0.85, and we find by lattice Boltzmann simulations that the solute transport process can be anomalous in all cases at high Péclet numbers. We use the continuous time random walk theory to quantitatively explain the observed scaling relations of the process. A plausible hydrodynamic origin of anomalous transport in simple porous media is proposed as a complement to its widely accepted geometric origin in complex porous media. Our results, together with previous findings, provide evidence that anomalous transport is indeed ubiquitous in porous media. Consequently, attentions should be paid when modelling solute transport by the classical advection-diffusion equation, which could lead to systematic error.

## Introduction

Anomalous (or non-Fickian) transport has been recognized as a common phenomenon in porous media whose complex pore space geometry strongly influences the flow and transport processes therein^1,2^. Many field and laboratory experiments, as well as pore-scale numerical simulations^3–19^, have confirmed the existence of a non-Gaussian solute concentration profile and an asymptotically nonlinear dependence of the concentration spatial variance (*M*_2_ as defined below) on time *t* (i.e., ${M}_{2} \sim {t}^{\beta }$ with *β* ≠ 1). These two typical features of anomalous transport are in great contrast with the prediction of the classical advection-diffusion equation (ADE). Various approaches have been proposed to model the anomalous transport process by taking into account a widely distributed heterogeneous velocity field^2,20–37^. It is perhaps natural to ascribe the heterogeneity of the velocity field, and the resultant anomalous transport, to the complex geometry of pore space, and it is indeed so in some cases. For example, anomalous transport can emerge due to the non-trivial complex structure (scale-free connection patterns) of the underlying network in which the transport process takes place^20^. Also, experimental as well as numerical evidence has shown that the type of the transport process can undergo qualitative changes as the pore space structure becomes more complex^9^. However, on the other hand, we also notice there has been experimental and numerical evidence that even for some structurally “simple” porous media, the solute transport process can be anomalous at high Péclet numbers^4,14,38,39^. In such cases, the porous media are regular or very weakly complex in void space geometry; there can hardly be any non-trivial spatial structure that is complex enough to induce a highly heterogeneous velocity field. Hence, the structural complexity does not seem to be the major source of the anomaly. Concerning these facts, one may wonder how much geometric complexity is needed to induce anomalous transport, and whether anomalous transport can be persistently observed in porous media with simple pore space structure.

To address these questions, we in this work study the transport process for a toy model of porous media that is constructed to be structurally as simple as possible. By doing so, one can exclude, or at lease greatly suppress, the influence of structural complexity on solute transport. While, at the mean time, the model still contains some minimal degree of randomness to make the results generalizable. Also, this model of porous media is similar to the setting in some laboratory experiments^4^. We then use the lattice Boltzmann method (LBM)^14,40–46^ to numerically solve the Navier-Stokes equations (NSEs) and the ADE in turn for each realization of the porous medium with a varying porosity *ϕ*. Scaling exponents of the concentration spatial moments (*M*_1,2_, see below) are calculated to compare with those obtained theoretically by the continuous time random walk (CTRW) theory.

We find that anomalous transport is astonishingly prevalent in our simple porous medium model when advection plays a dominant role. The anomaly exponent *β* ≠ 1 persists for porosities ranging from *ϕ* ≈ 0.30 up to *ϕ* ≈ 0.85. The observed scalings of *M*_1_ and *M*_2_ can be explained by the CTRW theory, which takes advantage of the statistics of the reciprocal steady-state velocity field, i.e., the statistics of 1/*u*, where *u* is the magnitude of the flow velocity **u**. In fact, *t* = 1/*u* is interpreted as the waiting time at Pe = ∞ in the CTRW theory. We use a mixture model to describe the probability density function (PDF) of *t*, denoted *w*(*t*). We argue conceptually it is helpful to decompose the pore-scale flow field into two distinct parts with the first being a globally uniform velocity field and the second being a locally fluctuating velocity field. Physically, the first part is essentially a manifestation of Darcy’s law and helps to explain the numerically observed linear scaling ${M}_{1} \sim t$; the second part gives rise to the anomalous feature of the transport process and controls the scaling of *M*_2_. We find $w(t) \sim {t}^{-1-\alpha }$ with 1 < *α* < 2 for the whole range of porosity considered in this work. The theoretical prediction that *β* = 3 − *α* is confirmed, which is an evident signature of anomalous transport.

The prevalence of anomalous transport in our simple model strongly indicates that such an anomaly is ubiquitous in general porous media. By showing an extreme case of anomalous transport in two dimensional Poiseuille flow, we further argue that the emergence of anomalous transport is a result of the joint action of hydrodynamics and pore space geometry. Qualitatively, the physical requirement of a no-slip boundary condition results in a quasi-parabolic velocity profile in throats as a solution to the NSEs. Combinations of throats constitute the main body of fluid pathways in porous media, which eventually lead to a heterogeneous flow field and hence anomalous transport^39^. For structurally simple porous media, hydrodynamics plays a crucial role in inducing non-uniform velocity profiles, while the influence of geometry manifests itself by enhancing the heterogeneity of the flow field as structural complexity grows. In our work, if we consider porosity as some “mean-field” measure of the pore space complexity, then we will see as the porosity *ϕ* is decreased, the exponent *α* follows, thus leading to a more heterogeneous flow field.

One implication of our results is that the conventional ADE on the Darcy scale may not be qualitatively adequate in describing transport dynamics in general porous media, due to the ubiquity of anomalous transport at high Péclet numbers. Thus, to avoid systematic error, approaches such as the CTRW theory may be adopted as more proper modelling tools^2^.

## Results

### Observation of anomalous transport in model porous media

 The methods to generate the porous medium and to simulate fluid flow and solute transport are detailed in Methods. After generating the desired porous medium and obtaining the steady-state fluid velocity field, we investigate how the solute concentration profile is changed with time. In particular, to quantify the statistical features of the profile, by which the anomalous nature of the process can directly be recognized, we calculate the first and second central moments of the concentration field in the *x* direction, denoted *M*_1_ and *M*_2_, respectively. Numerically, they are computed as *M*_1_ = *μ*_1_ and ${M}_{2}={\mu }_{2}-{\mu }_{1}^{2}$, respectively^3^, with ${\mu }_{1}=\frac{{\sum }_{i,j}\,c(i,j)x(i)}{{\sum }_{i,j}\,c(i,j)}$, and ${\mu }_{2}=\frac{{\sum }_{i,j}\,c(i,j){x}^{2}(i)}{{\sum }_{i,j}\,c(i,j)}$. Here *x*(*i*) denotes the *x* coordinate of cells with the first index being *i* and *c*(*i*, *j*) is the solute concentration at the cell indexed by *i* and *j*.

Extensive numerical simulations are performed to investigate how *M*_1_ and *M*_2_ behave as *t* is increased, at various *ϕ* and Pe. We mainly change *ϕ* from 0.30 to 0.85. We adopt Pe = 50 and Pe = 0.5 in our simulations to qualitatively represent two limiting situations: In the first case, advection is dominating; in the second case, the effect of diffusion is more important. An astonishing finding is that anomalous transport is unexpectedly prevalent in the first case, characterized by an anomaly exponent *β* ≠ 1 and ${M}_{2} \sim {t}^{\beta }$.

**Figure 1:**
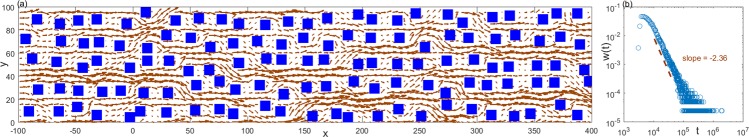
(**a**) The steady-state velocity field **u**(**x**) for a porous medium with *ϕ* ≈ 0.80; (**b**) the associated waiting time PDF *w*(*t*) at Pe = ∞ is obtained by statistics of *t* = 1/*u*, which displays a fat tail that scales as $\sim \,{t}^{-2.36}$ when *t* ∈ (10^4^, 10^5^). Throughout this work, the spacial resolution of the velocity field is five cells, i.e., the velocity at a position as represented by an arrow is plotted every five cells. The length of an arrow is proportional to the corresponding *u*.

**Figure 2:**
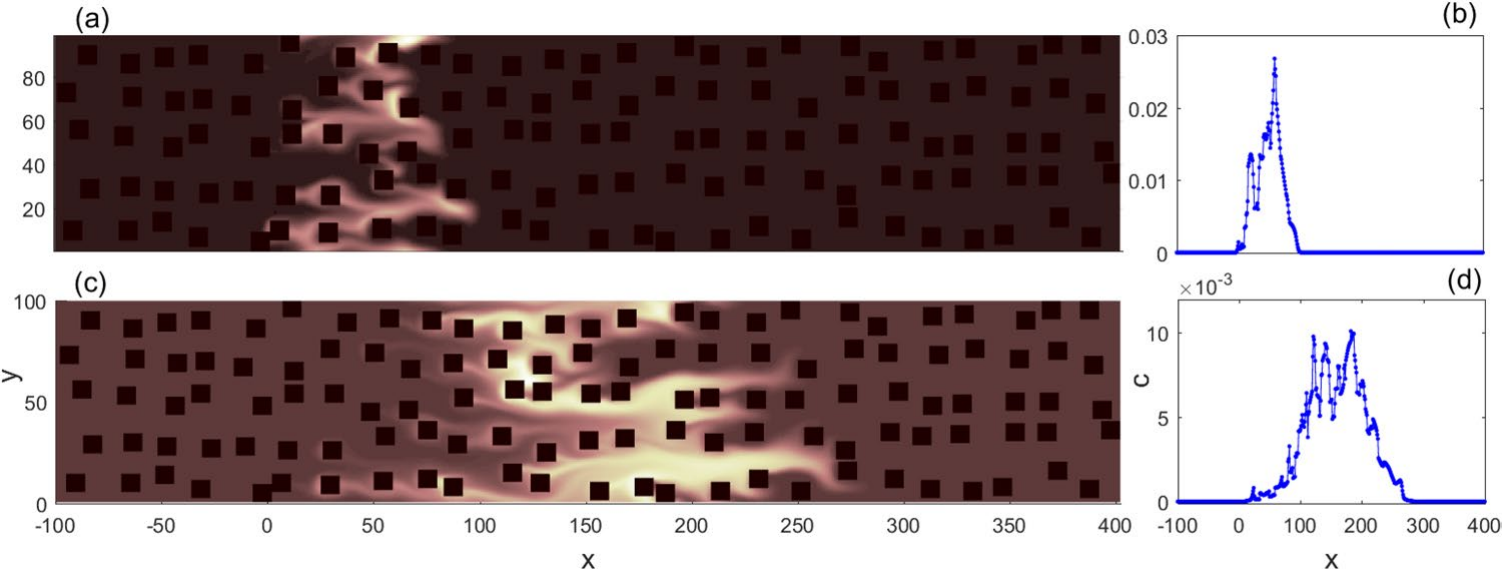
Pe = 50, for the same porous medium as in Fig. 1: (**a**) snapshot of the concentration profile at *t* = 0.1 × *t*_est_; (**b**) the corresponding cumulative concentration distribution in the *x* direction, *c*(*x*), at *t* = 0.1 × *t*_est_; (**c**) snapshot of the concentration profile at *t* = 0.3 × *t*_est_; (**d**) *c*(*x*) at *t* = 0.3 × *t*_est_. *t*_est_ roughly denotes the time for a solute particle with average velocity to reach the boundary (see Methods for its precise definition).

**Figure 3:**
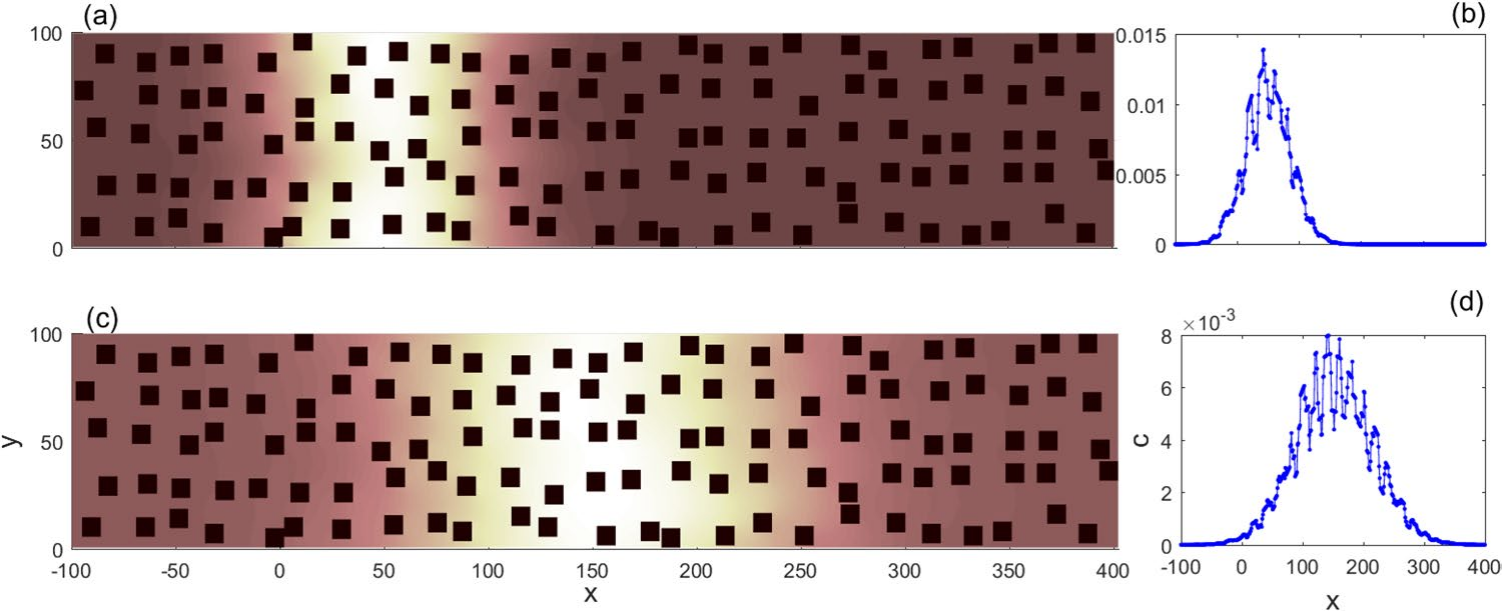
Pe = 0.5, for the same porous medium as in Fig. 1: (**a**) snapshot of the concentration profile at *t* = 0.1 × *t*_est_; (**b**) *c*(*x*) at *t* = 0.1 × *t*_est_; (**c**) snapshot of the concentration profile at *t* = 0.3 × *t*_est_; (**d**) *c*(*x*) at *t* = 0.3 × *t*_est_.

An illustrative example is presented in the following to show the emergence of anomalous transport in the advection dominant situation, in which the domain size is 500 × 100 and *ϕ* ≈ 0.8. Other parameters are: kinematic viscosity *ν* = 1.0, the density at the outlet *ρ*_east_ = 1.0, and the density drop Δ*ρ* = 0.01. *l* = 9 is used to generate the solid block. We in Fig. 1(a) plot the steady-state flow field in this case, whose spacial resolution is five cells, i.e., the fluid velocity, as denoted by the arrow, is plotted every five cells. The seemingly narrow channels, however, almost always contain enough cells to ensure a reliable LBM simulation^40^. The mean velocity in the *x* direction is ${\bar{u}}_{x}=9.2\times {10}^{-5}$, hence the Mach number is ${\rm{Ma}}=1.6\times {10}^{-4}\ll 1$. We also analyze the statistics of the fluid velocity field and plot in Fig. 1(b) the PDF *w*(*t*) of *t* ≡ Δ*x*/*u* = 1/*u*, where Δ*x* is the spacial step in LBM. We note that *w*(*t*) has a peak at some t^*^. For *t* < t^*^, *w*(*t*) drops drastically, while for *t* > t^*^, *w*(*t*) displays a fat tail, scaling as $w(t) \sim {t}^{-1-\alpha }$ with *α* ≈ 1.36 roughly on a time interval *t* ∈ (10^4^, 10^5^). If advection dominates the transport dynamics, then intuitively, this tail means solute particles may spend a much longer time at some cells than at others, and the solute particles are likely to move along the pressure drop direction at a non-uniform pace, demonstrating a spatially inhomogeneous concentration profile. However, when diffusion outweighs advection in affecting the transport process, a more homogeneous concentration field is expected to be observed. These are shown in Figs 2 and 3, where we plot the concentration fields for the case Pe = 50 (the corresponding diffusion coefficient *D*_m_ = 1.66 × 10^−5^) and Pe = 0.5 (*D*_m_ = 1.66 × 10^−3^), respectively.

In Fig. 2, we plot the concentration profiles, as well as the corresponding spatial distribution of accumulated concentration in the *x* direction $c(x(i))\equiv \sum _{j}c(i,j)$, at times 0.1 × *t*_est_ and 0.3 × *t*_est_, respectively. *t*_est_ roughly denotes the time for a solute particle with average velocity to reach the boundary (see Methods). The non-uniform concentration profile is evident. As advection is dominant, solute particles that move along some streamline to reach a stagnant zone ($u/{\bar{u}}_{x}\ll 1$ locally) will get stuck there and only have very low probability to escape. This is because the diffusion effect is too weak to induce effective particle transitions from such stagnant zones to regions where *u* is large via the transport along some adjacent streamline. Such suppression of particle transition between nearby streamlines is supposed to be the main reason why the solute concentration is distributed heterogeneously at high Pe.

However, when the diffusion effect is strong on transport, the spatial distribution of the concentration field is qualitatively different. In this situation, molecular diffusion will help solute particles hop out of stagnant zones, and overall the concentration profile will become spatially homogeneous, as shown in Fig. 3. Two snapshots of the concentration field for Pe = 0.5 at times 0.1 × *t*_est_ and 0.3 × *t*_est_ are also plotted, respectively, together with *c*(*x*). Compared with Fig. 2, it is even visually clear that solute particles are transported in a more uniform way. Note that *M*_2_ reflects the “spread” of the distribution of solute concentration in space, hence at a given time *t* a larger *M*_2_(*t*) quantitatively corresponds to a more homogeneous transport process than a smaller *M*_2_(*t*) does. At 0.1 × *t*_est_ or 0.3 × *t*_est_, *M*_2_ under Pe = 0.5 is way greater than that under Pe = 50 (Actually, the former is always greater than the latter in our simulations). That is to say, diffusion enhances the transport process. Although random hop rates between nearby streamlines may be the same^47^, the velocity fields along nearby streamlines are by no means always similar. Streamlines undergo a sudden “compression” when entering a narrow throat, which is a typical structure in porous media. In the outside wider region, the velocity gradient between nearby streamlines is much smaller than that within the throat. If a particle hops from one streamline which has a low velocity in the throat (near the solid boundary) to another streamline which has a high velocity in the throat (near the center of the throat), then it has high probability to be transported quickly forward, without hopping back to the slower streamline in the throat. On average, as long as the particles hop to the “express way,” they leave the stagnant zone in a short time. This is why diffusion speeds up the transport through throats. When particles are trapped within some region almost enclosed by solid cells, diffusion is the only possible mechanism for particles to get out. So, as the diffusion effect is increased, the concentration profile becomes more homogeneous.

**Figure 4:**
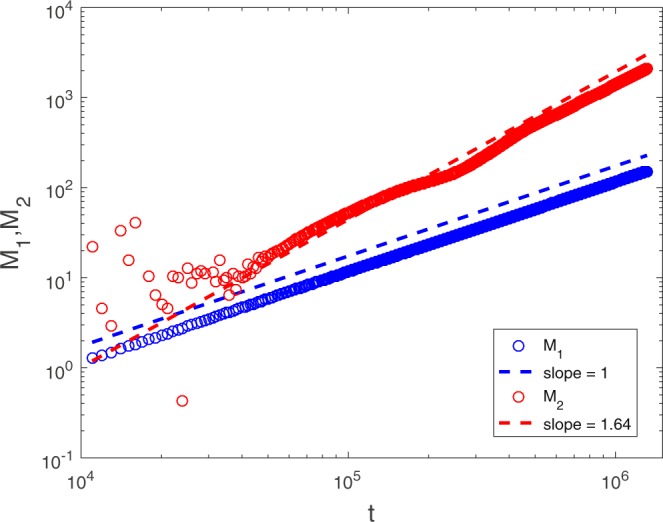
Pe = 50, and for the same porous medium as in Fig. 1, *M*_1_ and *M*_2_ are plotted versus time *t*. ${M}_{1} \sim t$ and ${M}_{2} \sim {t}^{\beta }$ with *β* = 1.64 can be observed approximately for *t* ∈ (4 × 10^4^, 4 × 10^5^). For *t* > 5 × 10^5^, *β* begins to deviate from 1.64.

**Figure 5:**
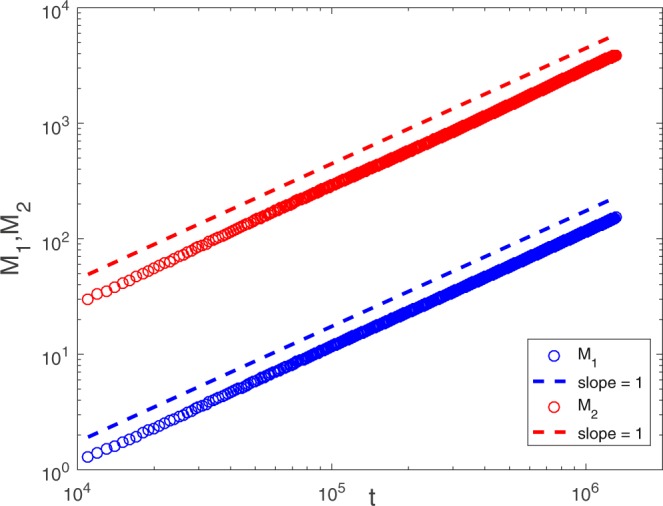
Pe = 0.5, and for the same porous medium as in Fig. 1, *M*_1_ and *M*_2_ are plotted versus time *t*. ${M}_{1} \sim t$ and ${M}_{2} \sim t$ can be observed.

**Figure 6:**
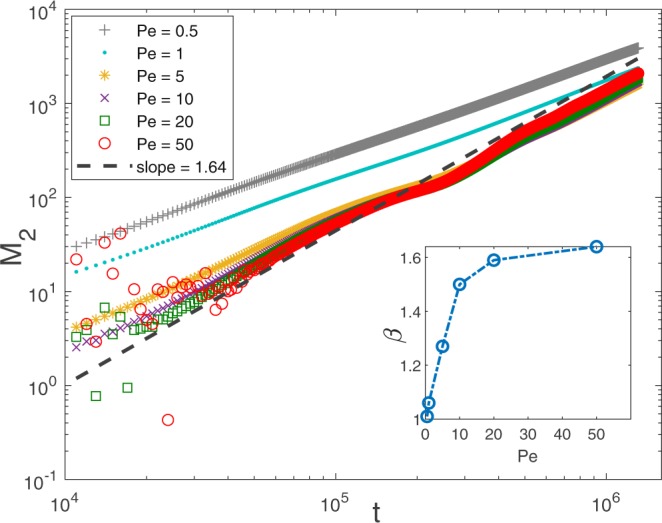
*M*_2_ under various choices of Pe are plotted as a function of time *t*. The porous medium in question is also the same as in Fig. 1. Inset shows how the scaling exponent *β* (in ${M}_{2} \sim {t}^{\beta }$) changes as Pe is increased.

Next, we investigate the transport process more quantitatively by tracking the time evolution of *M*_1_ and *M*_2_. In Figs 4 and 5, we plot how they evolve for Pe = 50 and Pe = 0.5, respectively. In the first case, we find that ${M}_{1} \sim t$, while ${M}_{2} \sim {t}^{\beta }$ with *β* = 1.64 for *t* ∈ (4 × 10^4^, 4 × 10^5^), which is a signature of anomalous transport. (But *β* seems to begin decreasing after *t* = 5 × 10^5^ due to the finiteness of Pe in simulation.) It is worth noting that there seems to be a quantitative relation between *w*(*t*) and *M*_2_ that *β* ≈ 3 − *α*. In the latter case of Pe = 0.5, since the concentration profile is rather “normal,” then not surprisingly, we find both *M*_1_ and *M*_2_ scale linearly with time, and the transport is Fickian. We also investigate the scaling behaviors of *M*_1_ and *M*_2_ under various choices of Pe, respectively. It turns out that ${M}_{1} \sim t$ is always present, whereas *M*_2_ will undergo a gradual transition from ${M}_{2} \sim t$ to ${M}_{2} \sim {t}^{1.64}$ as Pe is increased. We plot in Fig. 6 how *M*_2_ changes with time *t* as Pe is increased from 0.5 to 50. From Fig. 6, we can also see that when Pe is higher than 20, there are some numerical errors in the early stage of transport (inherent in the LBM when *τ*_c_ approaches 0.5, see Methods), however, the scaling of *M*_2_ is clearly not influenced at later times.

Actually, we have also found the scaling relations ${M}_{1} \sim t$ and ${M}_{2} \sim {t}^{3-\alpha }$ for many other realizations of the porous media with various *ϕ*. Anomalous transport characterized by such relations is thus prevalent at high Pe in our model, at least for some time interval *t* ∈ (*t*_lb_, *t*_ub_) with ${t}_{{\rm{ub}}}\approx {10}^{5} \sim {10}^{6}$ typically.

### Scaling relations at high Péclet numbers

In the CTRW theory, a particle hops from one site to another based on the transition rule that the transition distance is drawn from a PDF *λ*(**r**) and the waiting time between successive transitions is drawn from a PDF *w*(*t*). In our setting of the initial condition (see Methods), the master equation of the transition process reads

$$\begin{array}{lll}\eta ({\bf{x}},t) & = & \int d{\bf{x}}{\boldsymbol{^{\prime} }}{\int }_{0}^{{\rm{\infty }}}dt{\rm{^{\prime} }}\eta ({\bf{x}}{\rm{^{\prime} }},t)\lambda ({\bf{x}}-{\bf{x}}{\rm{^{\prime} }})w(t-t{\rm{^{\prime} }})\\  &  & +\sum _{j=1}^{m}\frac{1}{{n}_{{\rm{f}}}}\delta (x-x({i}_{0},j))(1-s({i}_{0},j))\delta (t),\end{array}$$

where *η*(**x**, *t*) is the PDF of a particle just arrives at **x** at time *t*. The PDF of finding a particle at **x** at time *t* is $W({\bf{x}},t)={\int }_{0}^{t}dt^{\prime} \eta ({\bf{x}},t^{\prime} ){\boldsymbol{\Psi }}(t-t^{\prime} )$, with ${\boldsymbol{\Psi }}(t)=1-{\int }_{0}^{t}\,w(t^{\prime} )dt^{\prime}$ being the probability that during a period of time *t*, a particle does not leave **x**. Since we are interested in the transport process in the direction of pressure drop, the vectorial **x** is simplified as *x* in the following, “intergarting out” the information in the *y* direction. By performing Fourier-Laplace transform to *W*(*x*, *t*)^2,31,32^, we obtain $W(k,z)=\frac{1-w(z)}{z}\frac{1}{1-\lambda (k)w(z)}$, where $\lambda (k)\equiv {\int }_{-\infty }^{\infty }\,{e}^{ikx}\lambda (x)dx$ is the Fourier transform of *λ*(*x*) and $w(z)\equiv {\int }_{0}^{\infty }\,{e}^{-zt}w(t)dt$ is the Laplace transform of *w*(*t*).

In this work, *λ*(*k*) is somewhat trivial, because a particle can only hop to adjacent cells, and ${\langle {x}^{n}\rangle }_{\lambda }\equiv {\int }_{-\infty }^{\infty }\,{x}^{n}\lambda (x)dx$ is finite for any non-negative integer *n*. Then as *k* → 0 we have *λ*(*k*) ≈ 1 + *ikl* − *k*^2^*σ*^2^/2, where *l* = 〈*x*〉_*λ*_ and *σ*^2^ = 〈*x*^2^〉_*λ*_. On the contrary, *w*(*z*) is non-trivial, we notice that *w*(*t*) in our work can be effectively written as a sum of two distributions:

1 $$w(t)\approx p{w}_{1}(t)+q{w}_{2}(t),$$

where *w*_1_(*t*) represents a distribution (for example, approximately a truncated Gaussian) that mainly accounts for the small-*t* part of *w*(*t*), *w*_2_(*t*) features the long-time tail of *w*(*t*), and *p*, *q* are some positive weighting parameters with *p* + *q* = 1. We are here not aiming at an accurate decomposition of *w*(*t*); by writing equation (1), we emphasize the fact that within the time interval of numerical simulations, neither *w*_1_ nor *w*_2_ is overwhelmingly dominant. Heuristically, *w*_1_(*t*) is well behaved in the sense that ${\langle t\rangle }_{1}\equiv {\int }_{0}^{\infty }\,t{w}_{1}(t)dt < \infty$, and ${w}_{1}(z)={\int }_{0}^{\infty }\,{e}^{-zt}{w}_{1}(t)dt\approx 1-{\langle t\rangle }_{1}z$ for *z* → 0. On the contrary, *w*_2_(*t*) is characterized by the *t*^−1−*α*^ tail with 1 < *α* < 2, and for small *z*, ${w}_{2}(z)={\int }_{0}^{\infty }\,{e}^{-zt}{w}_{2}(t)dt$ should have the property that 1 − *w*_2_(*z*) ≈ 〈*t*〉_2_*z* + *C*_*α*_*z*^*α*^ with ${\langle t\rangle }_{2}\equiv {\int }_{0}^{\infty }\,t{w}_{2}(t)dt < \infty$ and *C*_*α*_ being some constant^37^. Therefore, for small $z \sim 1/{t}_{{\rm{ub}}}\ll 1$, *w*(*z*) is approximated by

2 $$w(z)\approx 1-q{C}_{\alpha }{z}^{\alpha }-(p{\langle t\rangle }_{1}+q{\langle t\rangle }_{2})z.$$

Note that the *n*-th moment of *x*(*z*), which defines as $\langle {x}^{n}(z)\rangle \equiv {\int }_{-\infty }^{\infty }\,{x}^{n}W(x,z)dx$ with *W*(*x*, *z*) being the Laplace transform of *W*(*x*, *t*), can be obtained via the relation $\langle {x}^{n}(z)\rangle ={(-i)}^{n}{\frac{{\partial }^{n}W(k,z)}{\partial {k}^{n}}|}_{k=0}$^31^. Also note that ${\langle {x}^{n}\rangle }_{\lambda }={\mathrm{lim}}_{k\to 0}{(-i)}^{n}\frac{{d}^{n}\lambda (k)}{d{k}^{n}}$, then we have

3 $$\langle x(z)\rangle =-\,i{\frac{\partial W}{\partial k}|}_{k=0}=\frac{l}{z[1-w(z)]}.$$

According to equation (2), we rewrite 1 − *w*(*z*) as $1-w(z)\approx (p{\langle t\rangle }_{1}+q{\langle t\rangle }_{2})z(1+\frac{q{C}_{\alpha }}{p{\langle t\rangle }_{1}+q{\langle t\rangle }_{2}}{z}^{\alpha -1})$, and as a result

4 $$\begin{array}{rcl}\frac{1}{1-w(z)} & \approx  & \frac{1}{(p{\langle t\rangle }_{1}+q{\langle t\rangle }_{2})z}\frac{1}{1+\frac{q{C}_{\alpha }}{p{\langle t\rangle }_{1}+q{\langle t\rangle }_{2}}{z}^{\alpha -1}}\\  & \approx  & \frac{1}{(p{\langle t\rangle }_{1}+q{\langle t\rangle }_{2})z}(1-\frac{q{C}_{\alpha }}{p{\langle t\rangle }_{1}+q{\langle t\rangle }_{2}}{z}^{\alpha -1})\\  & \equiv  & \frac{A}{z}+\frac{B}{{z}^{2-\alpha }},\end{array}$$

where *A* = (*p*〈*t*〉_1_ + *q*〈*t*〉_2_)^−1^ and *B* = −*qC*_*α*_*A*^2^. Inserting equation (4) into equation (3), we find $\langle x(z)\rangle \approx \frac{lA}{{z}^{2}}+\frac{lB}{{z}^{3-\alpha }}$, whose inverse Laplace transform yields $\langle x(t)\rangle \approx lAt+\frac{lB}{{\rm{\Gamma }}(3-\alpha )}{t}^{2-\alpha }$, where Γ is the gamma function. In the long time limit, the term *lAt* dominates, hence

5 $${M}_{1}=\langle x(t)\rangle  \sim t\,(1 < \alpha  < 2)$$

is expected to be observed. Similarly, 〈*x*^2^(*z*)〉 can be obtained as $\langle {x}^{2}(z)\rangle \,=\,-\,{\frac{{\partial }^{2}W}{\partial {k}^{2}}|}_{k=0}\approx \frac{2{l}^{2}}{z{[1-w(z)]}^{2}}+\frac{{\sigma }^{2}}{z[1-w(z)]}$. Then also by equation (4), it is straightforward to obtain $\langle {x}^{2}(z)\rangle \approx \frac{2{l}^{2}{A}^{2}}{{z}^{3}}+\frac{4{l}^{2}AB}{{z}^{4-\alpha }}+\frac{2{l}^{2}{B}^{2}}{{z}^{5-2\alpha }}+\frac{{\sigma }^{2}A}{{z}^{2}}+\frac{{\sigma }^{2}B}{{z}^{3-\alpha }}$. Performing inverse Laplace transform to it, we find $\langle {x}^{2}(t)\rangle \approx \,{l}^{2}{A}^{2}{t}^{2}+\frac{4{l}^{2}AB}{{\rm{\Gamma }}(4-\alpha )}{t}^{3-\alpha }+\frac{2{l}^{2}{B}^{2}}{{\rm{\Gamma }}(5-2\alpha )}{t}^{4-2\alpha }+{\sigma }^{2}At+\frac{{\sigma }^{2}B}{{\rm{\Gamma }}(3-\alpha )}{t}^{2-\alpha }$. Combining the results for 〈*x*^2^(*t*)〉 and 〈*x*(*t*)〉, we arrive at $\langle {\rm{\Delta }}{x}^{2}(t)\rangle =\langle {x}^{2}(t)\rangle -{\langle x(t)\rangle }^{2}\approx \frac{\mathrm{2(}\alpha -\mathrm{1)}}{{\rm{\Gamma }}\mathrm{(4}-\alpha )}{l}^{2}AB{t}^{3-\alpha }+o({t}^{3-\alpha })$, and finally the scaling relation of *M*_2_ is obtained as

6 $${M}_{2}=\langle {\rm{\Delta }}{x}^{2}(t)\rangle  \sim {t}^{3-\alpha }(1 < \alpha  < 2).$$

Therefore, the scaling relations (5) and (6) are in accord with numerical results. It is worth noting that, however, such scalings are derived under the condition that $z \sim 1/{t}_{{\rm{ub}}}$, and they are only expected to be observed for time up to $t \sim {t}_{{\rm{ub}}}$. For $t\gg {t}_{{\rm{ub}}}$, since Pe is not strictly infinity, the diffusion process begins to take effect and the scalings for normal transport processes will emerge that ${M}_{\mathrm{1,2}} \sim t$. This may be the reason why there are deviations of *β* from 1.64 as time is increased in Fig. 4.

**Figure 7:**
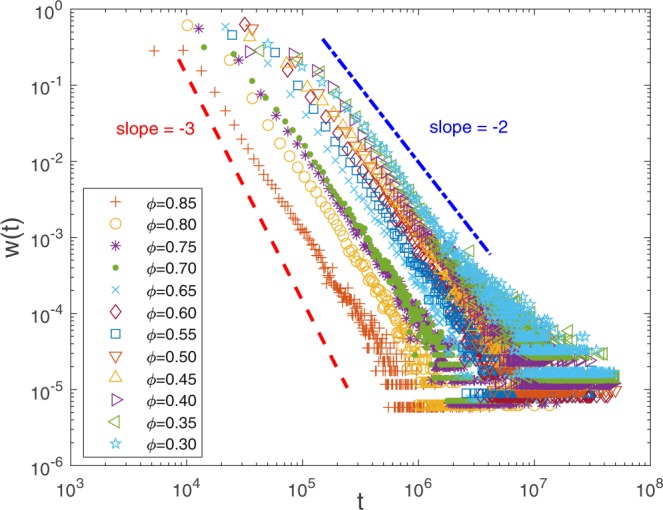
Typical waiting time distributions *w*(*t*) at Pe = ∞ are plotted for various porosities *ϕ* with the porous medium size 1000 × 200. Two lines with slopes −2 and −3, respectively, on a log-log scale are also plotted to guide eyes. For each case considered in this work, *w*(*t*) displays a fat tail $\sim \,{t}^{-1-\alpha }$ within some range of time, and the scaling exponent *α* is evidently in between 1 and 2, which is a signature of anomalous transport according to the continuous time random walk theory.

After establishing the relation between *α* and *β*, we can understand the prevalence of anomalous transport with the help of *w*(*t*). Keeping other major simulation parameters unchanged, we systematically investigate *w*(*t*) by varying porosities *ϕ* and solid block sizes *l* × *l*. In Fig. 7, typical *w*(*t*)’s are plotted for *ϕ* ∈ [0.30, 0.85] and *l* ∈ [9, 17]. In each case, *w*(*t*) approximately displays a fat tail $\sim \,{t}^{-1-\alpha }$. Also plotted are two lines with slope −2 and −3 on a log-log scale, respectively, to guide eyes. We can see 1 < *α* < 2 holds in all the cases we consider. In fact, according to the above theoretical work, as long as the decomposition of *w*(*t*) into two distinct parts is valid, then for any 1 < *α* < 2, the scaling relations (5) and (6) are expected to be observed. This implies that for a wide range of porosity, the transport dynamics at high Pe is anomalous, which originates from the fat tail of *w*(*t*). We also observe that *α* seems to decrease as *ϕ* is decreased. This reflects the influence of structure, since the porosity can be seen as a basic mean-field description of pore space geometry. In some sense, as *ϕ* is decreased, the pore space becomes more “complex,” and the transport process becomes more “anomalous.”

## Discussion

One issue we have not addressed is why *w*(*t*) typically displays a fat tail. The CTRW theory only takes advantage of this property to predict the scalings of anomalous transport, but itself does not provide any physical explanation why such a tail exists. At high Pe, *w*(*t*) is determined by the statistics of 1/*u*, and if we denote the PDF of *u* as *f*_*u*_(*u*), then *w*(*t*) = *f*_*u*_(1/*t*)|*du*/*dt*| = *f*_*u*_(1/*t*)*t*^−2^. So, as long as *f*_*u*_(*u*) is a mildly increasing function of *u* on some interval that can be well approximated by $\sim \,{u}^{\alpha -1}$, the *t*^−1−*α*^ scaling will be observed. This might suggest that the fat tail in *w*(*t*) is indeed not that unusual.

We may gain some insights from fluid mechanics about this point. It is a classical result^48^ that the fluid velocity near a solid disk with radius 1 is $u(r) \sim \sqrt{{\cos }^{2}\theta {(1-\frac{3}{2r}+\frac{1}{2{r}^{3}})}^{2}+{\sin }^{2}\theta {(1-\frac{3}{4r}-\frac{1}{4{r}^{3}})}^{2}}$, where *r* is the distance to the center of the solid disk, and *θ* is the angle with respect to the direction of the unperturbed incoming flow with velocity ${u}_{{\rm{i}}{\rm{n}}}$. The waiting time $t \sim 1/u$, and the long-time tail of *w*(*t*) is mainly contributed by $u/{u}_{{\rm{i}}{\rm{n}}}\ll 1$. Define *ε* = *r* − 1, then by expanding *u*(*r*) in the vicinity of *r* = 1, we have $1/t \sim \varepsilon +O({\varepsilon }^{2})$ for 0 < *θ* < *π*/2. Denote *f*_*ε*_(*ε*) as the PDF of *ε*. If a site is picked out at random and *θ* is uniformly distributed in (0, 2*π*), note that 1*dxdy* = *rdrdθ* = (1 + *ε*)*dεdθ*, then we have *f*_*ε*_(*ε*) ∝ (1 + *ε*), and consequently $w(t)={f}_{\varepsilon }(\varepsilon (t))|d\varepsilon /dt| \sim \,{t}^{-2}+O({t}^{-3})$.

**Figure 8:**
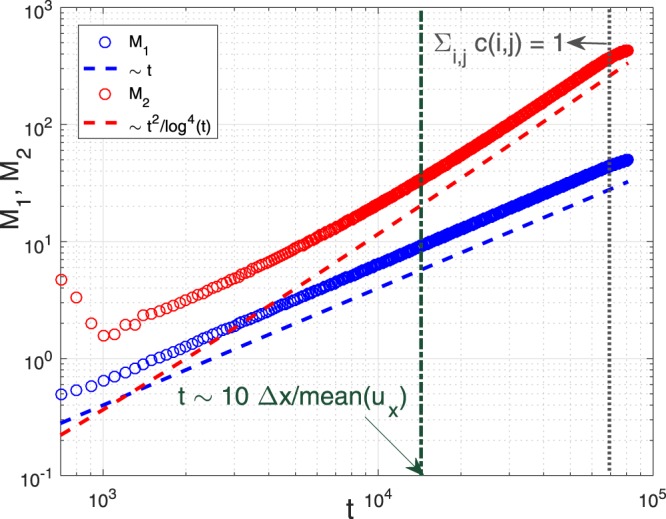
*M*_1_ and *M*_2_ are both in good agreement with theory. Pe = 100 and the solute concentration is kept as unity for a sufficiently long time after the predicted scaling behavior of *M*_2_ begins to emerge.

As another example, let us consider the Poiseuille flow in two dimensions. Qualitatively, the velocity profile perpendicular to the flow direction is $u(y) \sim 1-{y}^{2}$, where *y* is the normalized distance to the center of the throat. Then $t=1/u \sim {\mathrm{(1}-{y}^{2})}^{-1}$, and therefore $w(t) \sim {f}_{y}(y)|dy/dt|$, where *f*_*y*_(*y*) = 1 is the PDF of a randomly chosen *y*. We again find $w(t) \sim {t}^{-2}+O({t}^{-3})$. Quantitatively, we solve this problem using the LBM simulations. We also set Δ*x* = Δ*t* = 1 and the domain size *n* × *m* is 100 × 50. We choose *ν* = 1, *ρ*_east_ = 1, and Δ*ρ* = 0.001. The no-slip condition is applied to the north and south boundaries, and the constant-pressure condition is applied to the west and east boundaries when simulating the NSEs. While, we apply the adsorption condition to the west and east boundaries when numerically solving the ADE. As the steady-state is achieved, ${\bar{u}}_{x}=6.38\times {10}^{-4}$. Then, we uniformly distribute solute particles in the *y* direction, and they are transported under the joint action of advection and diffusion. We in this case define the Péclet number as ${\rm{Pe}}={\bar{u}}_{x}n/{D}_{{\rm{m}}}$. Following similar theoretical analysis as shown above, we have at Pe = ∞ that

7 $$w(t) \sim \frac{1}{{t}^{2}}\frac{{\rm{\Theta }}({u}_{0}t-\mathrm{1)}}{\sqrt{1-\frac{1}{{u}_{0}t}}},$$

where ${u}_{0}=1.5\times {\bar{u}}_{x}$ is the maximal *u*_*x*_ and Θ(*x*) denotes the Heaviside function which equals 1 when *x* > 0. Based on (7), one can see the average waiting time 〈*t*〉_*w*_ is finite and hence ${M}_{1} \sim t$. While for *M*_2_, note that as $t\gg 1/{u}_{0}$, $w(t) \sim {t}^{-2}$, then from the CTRW theory one expects ${M}_{2} \sim {t}^{2}{/\mathrm{log}}^{{\rm{4}}}(t)$^32^ when *t* is much more than 1/*u*_0_. In Fig. 8, Pe is set to be 100 and we clearly observe the good agreement between theory and simulations. In particular, *M*_2_ becomes well represented by the theoretical scaling behavior when $t \sim 10\times {\rm{\Delta }}x/{\bar{u}}_{x}{\mathrm{ > 10}}^{4}$.

From the two examples presented above, we can see even for “normal” situations in fluid mechanics, the fat tail of *w*(*t*) exists at high Pe. In our porous medium model, the flow confronting a solid block and between two solid blocks can be approximated by these two cases. The above analyses might still be qualitatively reasonable, and at least partially justify that the ubiquitous fat tail of *w*(*t*) is more of a hydrodynamic than a geometric origin. In a recent work^39^, a similar analysis was made to show quantitatively that anomalous transport is present in weakly complex porous media and can be predicted by incorporating the Poiseuille flow field with a set of throat sizes that follow a power-law distribution, consistent with our results. We thus conclude that, under the joint influence of hydrodynamics and geometry, anomalous transport is indeed ubiquitous in porous media, especially at high Pe.

In this work, we mainly focus on transport dynamics in model geological systems where the heterogeneous flow field in pore space gives rise to anomalous transport. The setting of our model is relevant in such fields as petroleum engineering and groundwater science. It is worth noting that aside from geological systems, anomalous diffusion is also observed in biological systems where, however, the main sources of the anomaly are the macromolecular complexity and spatiotemporal membrane heterogeneity^49^. Geometry-induced anomalous diffusion can be found in other cases as well^50,51^. In many biological systems, the spatial scale of interest is typically ${10}^{-9} \sim {10}^{-6}\,{\rm{m}}$, and experiments as well as numerical simulations can be performed using the single particle tracking technique to accurately capture each particle’s dynamics. As a result, important statistics of a single-particle trajectory can be obtained for analyzing the *time-averaged* mean squared displacement. While in geological systems, although simulations can be done for rock samples of size $\sim \,{10}^{-6}\,{\rm{m}}$, laboratory experiments can only be performed on rock samples of size $\sim \,{10}^{-1}\,{\rm{m}}$, and the real transport process of engineering interest takes place on a spatial scale $\sim \,{10}^{3}\,{\rm{m}}$. Hence, a macroscopic *ensemble-averaged* description of the process by CTRW modelling or a fractional ADE is perhaps more preferred in this sense. In this work, by using the LBM to directly simulate the evolution of the concentration field (equivalent to the single-particle PDF), we deal with the particle ensemble from the very beginning and neglect information such as the two-point time correlation. Consequently, our CTRW analysis only uses the most basic model and does not address issues like weak ergodicity breaking and ageing^52–54^, which are important in the analysis of anomalous diffusion in biological systems and surely worth investigating in geological systems as well. However, that will involve numerical algorithms totally different than the LBM, and we wish to leave these for future study.

## Methods

### Generation of Porous Media

A porous medium in this work is composed of *n* × *m* cells; each is of unit size. The state of a cell indexed by (*i*, *j*) with *i* = 1, …, *n* and *j* = 1, …, *m* is denoted *s*(*i*, *j*) with *s*(*i*, *j*) = 1 for a solid cell and *s*(*i*, *j*) = 0 for a fluid cell. To achieve a statistically homogeneous porous medium, we divide the *n* × *m* region into ${n}_{{\rm{S}}}=\frac{n}{20}\times \frac{m}{20}$ subregions, each of the size 20 × 20. In every subregion, a solid block consisting of *l* × *l* solid cells is generated randomly in position with probability *ϕ*/(1 − *l*^2^/*n*_S_). For a given *l*, the minimum achievable porosity is 1 − *l*^2^/*n*_S_. By adjusting *l* from 9 to 17, we generate porous media with porosities ranging from roughly $0.30 \sim 0.85$ in this work. Adopting *l* in this range also ensures the Knudsen number Kn to be small so that the LBM simulation results are reliable^40^. After generating a porous medium from some given porosity *ϕ*, we calculate the realized porosity *ϕ*_real_ = 1 − *N*_s_/*nm*, where ${N}_{{\rm{s}}}={\sum }_{i,j}\,s(i,j)$ is the number of solid cells. If |*ϕ*_real_ − *ϕ*| < Δ*ϕ*_th_, where Δ*ϕ*_th_ is a prescribed threshold and is chosen to be less than 0.03 in our simulation, we will proceed with the generated porous medium; otherwise, we will re-sample until the criterion is met.

By the generating rule above, the porous media thus constructed are statistically homogeneous. We use such a simplest model to suppress the structural effect on transport as much as possible, while at the mean time a minimal level of randomness is maintained to make the results more generalizable.

### Simulation of Fluid Flow

Once the porous medium is generated, we numerically solve the NSEs by the standard single relaxation time D2Q9 scheme of the LBM^14,40,41^:$${f}_{i}({\bf{x}}+{\rm{\Delta }}t{\xi }_{i},t+{\rm{\Delta }}t)={f}_{i}({\bf{x}},t)+\frac{1}{\tau }[{f}_{i}^{{\rm{eq}}}({\bf{x}},t)-{f}_{i}({\bf{x}},t)]$$, where the subscript *i* = 0, …, 8, denoting the *i* th direction, *f*_*i*_ is the single-particle distribution function, $${f}_{i}^{{\rm{eq}}}$$ is the local equilibrium distribution function, *τ* is the dimensionless relaxation time, Δ*t* = 1 is the time step used in this work, and *ξ*_*i*_ is the discrete velocity with *ξ*_0_ = (0, 0)^*T*^, *ξ*_*i*_ = (cos((*i* − 1)*π*/2), sin((*i* − 1)*π*/2))^*T*^ for *i* = 1, 2, 3, 4, and $${\xi }_{i}=\sqrt{2}{(\cos (\pi /4+(i-\mathrm{5)}\pi /\mathrm{2),}\sin (\pi /4+(i-\mathrm{5)}\pi /\mathrm{2))}}^{T}$$ for *i* = 5, 6, 7, 8. We also choose^43^ $${f}_{i}^{{\rm{eq}}}={\omega }_{i}[\rho +$$$$3{\xi }_{i}\cdot {\bf{U}}+\mathrm{4.5(}{\xi }_{i}\cdot {\bf{U}}{)}^{2}-1.5{U}^{2}]$$, where *ω*_*i*_′s are weighting factors (*ω*_0_ = 4/9, *ω*_1,2,3,4_ = 1/9, and *ω*_5,6,7,8_ = 1/36), *ρ* is the fluid density, and **U** = *ρ***u** is the mass current of the fluid, with **u** being the fluid velocity. *ρ* and **U** are calculated as $$\rho ={\sum }_{i=0}^{8}\,{f}_{i}={\sum }_{i=0}^{8}\,{f}_{i}^{{\rm{eq}}}$$, and $${\bf{U}}={\sum }_{i\mathrm{=0}}^{8}\,{f}_{i}{\xi }_{i}={\sum }_{i\mathrm{=0}}^{8}\,{f}_{i}^{{\rm{eq}}}{\xi }_{i}$$, respectively. With the above settings, one can obtain in the small Knudsen and Mach numbers limit^43^ that $$\frac{\partial \rho }{\partial t}+\nabla \cdot {\bf{U}}=0$$ and $$\frac{\partial {\bf{U}}}{\partial t}+{\bf{U}}\cdot \nabla {\bf{U}}=-\,\nabla p+\nu {\nabla }^{2}{\bf{U}}$$, where *ν* = (*τ* − 0.5)/3 is the kinematic viscosity, and the pressure *p* = *ρ*/3. Up to second order accuracy, this set of equations is equivalent to the classical NSEs: $$\nabla \cdot {\bf{u}}=0$$, and $$\frac{\partial {\bf{u}}}{\partial t}+{\bf{u}}\cdot \nabla {\bf{u}}=-\,\frac{\nabla p}{\rho }+\nu {\nabla }^{2}{\bf{u}}$$.

In the two-dimensional domain, boundary conditions are set as in a previous work^14^. We keep a constant pressure difference Δ*p* between west (inlet) and east (outlet) boundaries; this is numerically realized by keeping a constant density difference Δ*ρ*^44^, due to the linear dependence of *p* on *ρ*. North and south boundary conditions are periodic. The no-slip condition is applied to the interface between fluid and solid cells with a second order accurate bounce-back method^45^. Initially, the fluid is set to be at rest. After performing the LBM simulation for a transient period of time, we obtain a steady-state velocity field; the steady state is considered to be reached when the criterion $\frac{{\sum }_{{\bf{x}}}\Vert {\bf{u}}({\bf{x}},t+\mathrm{100)}-{\bf{u}}({\bf{x}},t)\Vert }{{\sum }_{{\bf{x}}}\Vert {\bf{u}}({\bf{x}},t)\Vert } < {10}^{-5}$ is satisfied. We let **u** = 0 for all solid cells. The Mach number is defined as ${\rm{Ma}}={\bar{u}}_{x}/{c}_{{\rm{s}}}$, where ${\bar{u}}_{x}$ is the average steady-state fluid velocity in magnitude in the direction of pressure drop and ${c}_{{\rm{s}}}=1/\sqrt{3}$ is the speed of sound in the D2Q9 scheme. We also define the Reynolds number ${\rm{Re}}={\bar{u}}_{x}n/\nu$. Both Ma and Re are small enough (${\rm{Ma}},{\rm{Re}}\ll 1$) to ensure the validity of the LBM simulation.

### Simulation of Solute Transport

Having obtained the steady-state velocity field, we proceed to simulate the solute transport process, which is also done by the LBM with a single relaxation time D2Q5 scheme^14^: ${g}_{i}({\bf{x}}+{\rm{\Delta }}t{\xi }_{i},t+{\rm{\Delta }}t)={g}_{i}({\bf{x}},t)+\frac{1}{{\tau }_{{\rm{c}}}}[{g}_{i}^{{\rm{eq}}}({\bf{x}},t)-{g}_{i}({\bf{x}},t)]$, where *i* = 0, …, 4, denoting the *i* th direction, *g*_*i*_ is the single particle distribution function, ${g}_{i}^{{\rm{eq}}}$ is the local equilibrium distribution function, *τ*_c_ is another dimensionless relaxation time. ${g}_{i}^{{\rm{eq}}}$ is chosen as^14^ ${g}_{i}^{{\rm{eq}}}={\tilde{\omega }}_{i}c\mathrm{(1}+2.5{\xi }_{i}\cdot {\bf{u}})$, where the weighting coefficient ${\tilde{\omega }}_{i}=1/5$ for all *i*′s. Then the solute concentration *c* is calculated as $c={\sum }_{i=0}^{4}\,{g}_{i}({\bf{x}},t)={\sum }_{i=0}^{4}\,{g}_{i}^{{\rm{eq}}}({\bf{x}},t)$. This numerical scheme in the small Knudsen number limit leads to^14^ $\frac{\partial c}{\partial t}+\nabla \cdot ({\bf{u}}c)={D}_{{\rm{m}}}{\nabla }^{2}c$, where *D*_m_ = (*τ*_c_ − 0.5)/2.5 is the molecular diffusion coefficient. Concerning the incompressibility condition ▽⋅**u** = 0, this equation is essentially the conventional ADE: $\frac{\partial c}{\partial t}+{\bf{u}}\cdot \nabla c={D}_{{\rm{m}}}{\nabla }^{2}c$.

In our simulation, *c* is normalized, i.e., ${\sum }_{i,j}\,c(i,j)=1$ at time *t* = 0, where *c*(*i*, *j*) denotes the solute concentration at a cell indexed by (*i*, *j*). The solute is initially placed uniformly at fluid cells that are on some vertical line *x* = *x*(*i*_0_). That is to say, *c*(*i*_0_, *j*) = (1 − *s*(*i*_0_, *j*))/*n*_f_ for all *j* = 1, …, *m*, with ${n}_{{\rm{f}}}={\sum }_{j}\,\mathrm{(1}-s({i}_{0},j))$ being the number of fluid cells on the line *x* = *x*(*i*_0_). Depending on the specific realization of the porous medium and the steady-state velocity field, *i*_0_ is in between 1 and *n*, typically chosen as 0.2*n*. And we choose *x*(*i*_0_) as the origin of the *x*-axis when calculating the moments of concentration.

As for boundary conditions, both north and south boundaries are set as periodic. The adsorption condition is applied to the west and east boundaries by adopting the zero concentration-gradient method^44^. Since our simulation results are to be interpreted according to the analytical solution of a CTRW model in which particles are transported in an infinite space, implying the summation of the normalized concentration always equals 1, we terminate the simulation process as long as one of the following two conditions is satisfied: ${\sum }_{i,j}\,c(i,j) < 0.95$, or the time exceeds 0.5 × *t*_est_ with ${t}_{{\rm{est}}}=(x(n)-x({i}_{0}))/{\bar{u}}_{x}$ being the time scale roughly equals to the time the concentration peak needs to move from *x*(*i*_0_) to the east boundary *x*(*n*). (Indeed, within this period of time, a dominant majority of solute concentration is kept in the computational domain, and ${\sum }_{i,j}\,c(i,j) > 0.95$ is almost always guaranteed in our simulations.) The solid cells are impermeable^44^ and the concentration *c* of a solid cell is set to be 0. Then, we define the Péclet number as ${\rm{Pe}}={\bar{u}}_{x}l/{D}_{{\rm{m}}}$. In simulations, however, we set the value of Pe first, and then derive the corresponding *D*_m_, which is fed into the program.

We observe that the LBM will show some numerical errors in the early stage of the solute transport process when Pe → ∞, or equivalently, when *D*_m_ is small. This is a known fact about the LBM as *τ*_c_ approaches 0.5. However, our algorithm is stable after the transient relaxation^55^. Also, we do not care much about the early stage of the transport process. The intermediate to long term scaling relations of *M*_1_ and *M*_2_, which are of primary interest in this work, do not suffer from such numerical errors, as shown in our examples.

Finally, using the LBM to solve the ADE is mathematically equivalent to solving a Fokker-Planck equation that describes the evolution of the single-particle PDF. One can compute the spatial moments like *M*_1_ and *M*_2_ at a given time *t*, however, a limitation is that the trajectory of a single particle cannot be tracked, hence a time-averaged quantity cannot be computed. In cases where such information is crucial, for example in the study of ageing, other methods like molecular simulations or Monte Carlo methods are necessary^49–53^, while typical computational fluid dynamics methods cannot achieve this goal.
